# The effect of initiating neuraxial analgesia service on the rate of cesarean delivery in Hubei, China: a 16-month retrospective study

**DOI:** 10.1186/s12884-020-03294-z

**Published:** 2020-10-12

**Authors:** Yun Zhao, Ying Gao, Guoqiang Sun, Ling Yu, Ying Lin

**Affiliations:** 1 Department of Obstetrics, Maternal and Child Health Hospital of Hubei Province, Tongji Medical College, Huazhong University of Science and Technology, Wuhan, 430070 China; 2 Department of Anesthesiology Maternal and Child Health Hospital of Hubei Province, Tongji Medical College, Huazhong University of Science and Technology, Wuhan, 430070 China

**Keywords:** Neuraxial labor analgesia, Cesarean delivery, Maternal request cesarean delivery

## Abstract

**Background:**

No Pain Labor &Delivery (NPLD) is a nongovernmental project to increase access to safe neuraxial analgesia through specialized training. This study explores the change in overall cesarean delivery (CD) rate and maternal request CD(MRCD) rate in our hospital after the initiation of neuraxial analgesia service (NA).

**Methods:**

NA was initiated in May 1st 2015 by the help of NPLD. Since then, the application of NA became a routine operation in our hospital, and every parturient can choose to use NA or not. The monthly rates of NA, CD, MRCD, multiparous women, intrapartum CD, episiotomy, postpartum hemorrhage (PPH), operative vaginal delivery and neonatal asphyxia were analyzed from January 2015 to April 2016.

**Results:**

The rate of NA in our hospital was getting increasingly higher from 26.1% in May 2015 to 44.6% in April 2016 (*p <* 0.001); the rate of CD was 48.1% (3577/7360) and stable from January to May 2015 (*p*>0.05), then decreased from 50.4% in May 2015 to 36.3% in April 2016 (*p <* 0.001); the rate of MRCD was 11.4% (406/3577) and also stable from January to May 2015 (*p*>0.05), then decreased from 10.8% in May 2015 to 5.7% in April 2016 (*p <* 0.001). At the same time, the rate of multiparous women remained unchanged during the 16 month of observation (*p*>0.05). There was a negative correlation between the rate of NA and rate of overall CD, r = − 0.782 (95%CI [− 0.948, − 0.534], *p*<0.001), and between the utilization rate of NA and rate of MRCD, r = − 0.914 (95%CI [− 0.989, − 0.766], *p*<0.001). The rates of episiotomy, PPH, operative vaginal delivery and neonatal asphyxia in women who underwent vaginal delivery as well as the rates of intrapartum CD, neonatal asphyxia, and PPH in women who underwent CD remained unchanged, and there was no correlation between the rate of NA and anyone of those rates from January 1st 2015 to April 30th 2016 (*p*>0.05).

**Conclusions:**

Our study shows that the rates of CD and MRCD in our department were significantly decreased from May 1st 2015 to April 30th 2016, which may be due to the increasing use of NA during vaginal delivery with the help of NPLD.

## Background

The rate of cesarean delivery (CD) in China [1–5] has risen markedly from 2.0% (14/701) in 1978–1985 to 36.6% (813/2224) during 2006–2010 [1]. In recent years, nearly half of all newborns in China were delivered by CD [2, 3], among which 23.2% were performed based on maternal request rather than on medical indications [6]. The high rate of overall CD may result in increased risk of maternal complications such as infection, hemorrhage, or even death [6]. Previous researches have showed that Chinese women often choose CD to avoid the pain during vaginal delivery, because normally no analgesia service will be given during their labor and delivery in China [2, 7]. Neuraxial analgesia (NA) for labor is a safe and commonly available method in developed countries [7], which has been recommended as a proactive approach for high-risk parturient during labor [8]. However, very few hospitals in China regularly carry out such approach at present [3, 4]. A high CD rate and low or no availability of NA for labor is a common situation in hospitals of China. No Pain Labor&Delivery(NPLD) is a nongovernment project, which was proposed by Ling-Qun Hu, an anesthesiologist at the Northwestern University Feinberg School of Medicine in 2008. After 10 years of efforts, this project has been supported by many nongovernment organizations from both America and China [7]. Before the use of NA, common services provided in our delivery room include midwives service, accompanying family members, and water immersion service [9] during the first stage of labor if patients had requirement (about 20% of them choose water immersion delivery). NA has been regularly carried out in our hospital since May 1st, 2015.

This study is a retrospective study based on a large birth center to evaluate the correlation between NA availability and the rates of overall CD, MRCD, episiotomy, postpartum hemorrhage (PPH), operative vaginal delivery and neonatal asphyxia in women who undergo vaginal delivery, and intrapartum CD and neonatal asphyxia in women who undergo cesarean delivery after regular implementation of NA. The data from January 1st, 2015 to April 30th, 2016 were collected from our hospital.

## Methods

### Participants and methods

This study was conducted from January 1st, 2015 to April 30th, 2016 in our hospital, a tertiary- care teaching hospital in Hubei province, in central China.

#### Ethical approval

The study protocol was retrospective research and was approved by the ethics committee of Maternal and Child Health Hospital of Hubei Province.

#### Preparation stage of NA in our hospital

On March 1st, 2015, an organizing committee of NA was founded, which consisted of 10 members from our hospital, including 1 administrator, 2 anesthesiologists, 1 obstetrician, 1 neonatologist, 1 nurse, 1 midwife, and 3 experts from NPLD team. Among the members, three experts were born and educated in China and then immigrated to the United States for further academic development. They were fluent in both Mandarin and English and familiar with Western standards of obstetric care. In our hospital, a meeting is regularly organized 1–2 times a week. Questions would be discussed and communicated among the experts from NPLD through WeChat (the most popular online chat platform in China designed by Tencent Company), and the care givers from our hospital had more than 5 years of work experience in the department of anesthesia (attending physician) and NPLD experts took at least 7 days for field guidance in our hospital.

Before May 1st, 2015, NA was not available in labor and delivery suite. All pregnant women with labor onset were generally transferred to labor and delivery suite when the dilation of cervix was more than 1 cm. If intrapartum CD was needed in labor, they would be transferred to the operating suite. In our labor and delivery suite, the maternal service mode was provided, including family member accompanying, various positions allowed, Doula, midwives during labor. However, during labor, no NA could be provided, and nearly 20% of them chose water immersion during first stage of labor. All of the parturient women received bilateral perineal block anesthesia when the fetal head was crowned. After delivery, all of postpartum women would be returned to maternity room again.

After May 1st, 2015, with the help of NPLD team via WeChat, NA could be provided every day at any time in our labor and delivery suite. One of our labor and delivery suites (total 10 rooms) was also set as operative suite to deal with maternal and fetal emergencies for extremely dangerous to undergo intrapartum CD, with an anesthesiologist and an anesthesia nurse on duty every day, two shifts a day, at 8:00 am and 17:00 pm, respectively.

#### The NPLD program in our hospital

From June 21 to 27 of 2015, a professional NPLD team consisting of 12 members from America (Supplementary file 1) travelled to our hospital to give us a one-week training [7]. The training was conducted in a typical “hands-on” pattern. First, the trainer from the team gave an overall introduction about the training, and then trainees (Chinese medical staff) practiced the drills, and finally multidisciplinary debriefings were held at the end of each day. The practiced drills included 5-min crash CD to deal with maternal and fetal emergencies for extremely dangerous, how to deal with neurological complications, how to use operative instrument, how to reduce the rate of episiotomy, and how to perform resuscitation of neonatal asphyxia.

#### Data sources

**Fig. 1:**
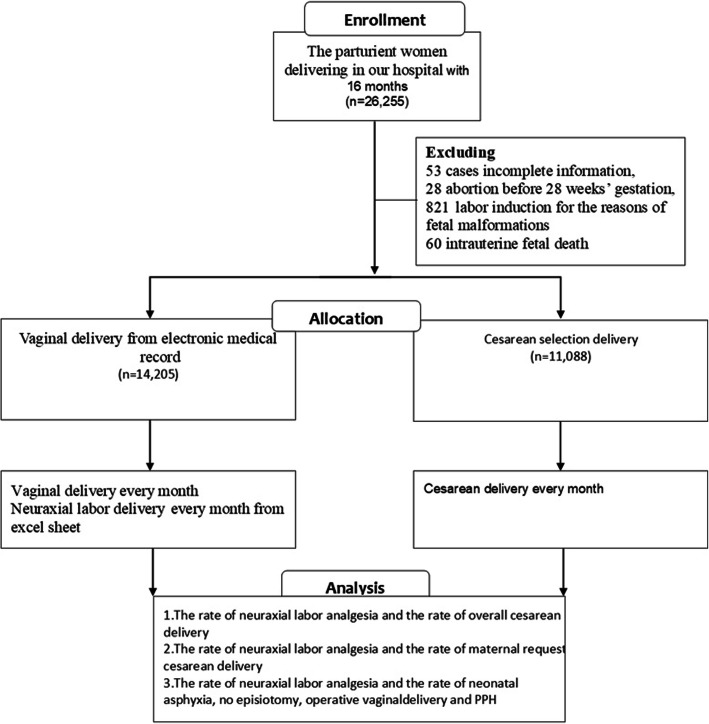
Flow Diagram

This retrospective study was based on the maternity departments of a tertiary- level public hospital in Wuhan, China. This is a large birth center, with annual number of newborn babies of around 20.000 in the most recent 3 years. The delivery data were collected from the hospital’s information system from January 1st, 2015 to April 30 th, 2016. A total of 26,255 deliveries were included in our study, of which 53 cases of incomplete information, 28 cases of abortion before 28 weeks’ gestation, 821 cases of labor induction for fetal malformation, 60 cases of intrauterine fetal death were excluded. Therefore, a complete dataset of 25,293 cases was finally included (accounting for 96.3% of total data). Among them, 5829 cases were from January 1st 2015 to April 30th 2015 before the implement of NA and 19,464 cases were from May 1st, 2015 to April 30th, 2016 after NA. The data contains information such as demographic data, mother’s age, gravidity, parity, date of delivery, principal diagnosis of maternal or fetal pregnancy-related complications, gestational age at delivery, mode of delivery, primary indications of CD, maternal request cesarean delivery (MRCD), intrapartum CD, whether or not using NA, episiotomy, postpartum hemorrhage (PPH), operative vaginal delivery, neonatal asphyxia (See Fig. 1).

In China, CD with medical indications can be divided into CD with absolute medical indications and CD with relative medical indications (for instance age more than 35 years). In our study, MRCD refers to a cesarean delivery based on maternal request, which lacks absolute or relative medical indications according to the classification standards above [10, 11].

#### Neuraxial labor analgesia

Women undergoing vaginal delivery were evaluated by anesthesiologist and obstetrician to assess their desire for and suitability for NA once their cervical dilation was greater than 1 cm. Parturient women who had any systemic and local sepsis, deranged coagulation profile, or drug allergy (Lidocaine, bupivacaine and fentanyl) were excluded from the study. For labor NA, following conditions need to be recorded: nulliparous or parous, spontaneous labor or medicine induced labor, full-term delivery or premature delivery, spontaneous vaginal delivery or instrument assisted vaginal delivery or intrapartum CD.

Epidural analgesia was initiated in the left lateral decubitus position. A sterile preparation with 1% alcohol iodophor was applied by an anesthesiologist after at least 500 mL of Ringer’s lactate solution was administered by an anesthesia nurse. Then, a 20-gauge catheter via a 17-gauge needle was inserted into the epidural space at the L3–4 or L4–5 interspace. Then 2–3 mL of 2% lidocaine was applied before observing for 5 min to see if there was any adverse reaction. If no adverse reaction was observed, the catheter was fixed and connected to a programmed intermittent epidural bolus (PIEB) pump (Master PCA pump, Fresenius Kabi USA, without continuous background infusion) to give 10 mL of mixed drugs of 0.08% ropivacaine +2μg/mL fentanyl. The PIEB pump was set to an automatic mode to deliver 10 mL of the drug mixture every 1 h. The patient could push the PIEB button whenever she felt uncomfortable or any pain during delivery and labor. Each press releases 10 mL of the drug mixture, but the interval between the two doses must be at least 15 min. The continuous infusion rate was set to 0 mL/h. Generally, anesthesia infusion was stopped after finishing the perineal stitch and was removed in 2 h after delivery.

Maternal HR, NIBP and SpO2 as well as fetal heart rate were continuously monitored throughout the PIEB period. Each parturient woman was supervised by a specialized midwife, and observed by an anesthesia nurse at regular intervals. Common side effects such as nausea, somnolence, and pruritus were recorded by anesthesia nurses. The PIEB pump was administered by the parturient woman herself according to the instruction of the anesthesiologist. Parturient women received exogenous oxytocin to obtain an enhanced labor process when indicated. All fetal and maternal events, therapeutic interventions, outcome of labor, and Apgar score of newborns at 1 and 5 min, PPH, and the mode of delivery were recorded. The analgesia effect was evaluated by visual analog scale (VAS) and numerical rating scale (NRS). The VAS was assessed on a 10 cm horizontal line. The selected pregnant women were informed that the left end of the scale represents “no pain” and that the right end represents the “most severe pain imaginable”. Then, they were instructed to mark the intensity of pain they were currently experiencing. For the NRS, a 11-point scale was used, with “0” representing “no pain” and “10” representing the “most severe pain imaginable.” All pain assessments were performed by an anesthesiologist before and after NA. After delivery, a questionnaire survey on the satisfaction on the NA effect was carried out by scanning the code on WeChat.

#### Patient’s education

All our patients were educated by obstetrician in prenatal examination and the brochure for NA were given to them. On working day, midwives conducted specialized classes about NA to teach them how to improve compliance during analgesia. Women undergoing vaginal delivery were evaluated by anesthesiologist and obstetrician to assess their desire for and suitability for NA once their cervical dilation was greater than 1 cm and all of them signed written informed consent for NA. Women undergoing vaginal delivery could also choose water immersion delivery once their cervical dilation was greater than 5 cm.

#### Data analysis

All data were inputted into SPSS software (v.19.0, SPSS Inc., Chicago, IL, USA) for statistical analysis. The Pearson correlation was adopted to evaluate the relationship among observed rates during 12 months. Value r and their 95% CIs were calculated. Cochran- Armitage Trend Test was carried out to evaluate the change trend of observed rates. All statistical tests were performed with 2-sided *P* values. If *p* value<0.05, the difference was considered statistically significant.

## Results

**Fig. 2:**
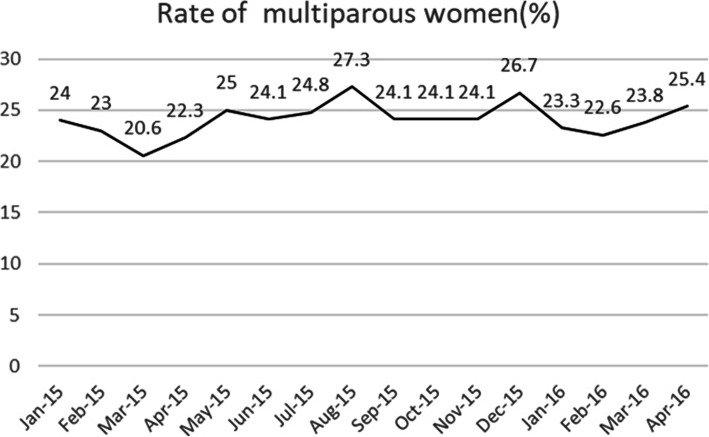
Rate of multiparous women

A total of 25,293 cases of parturient women were finally enrolled in our hospital from January 1st, 2015 to April 30th, 2016, of which 14,205 cases (56.2%) underwent vaginal delivery and 11,088 cases (43.8%) underwent CD. There were 19,196 (75.9%) nulliparous and 6097 (24.1%) parous, and the rate of parous remained unchanged over the 16-month study period according to Cochran- Armitage Trend Test (z = 3.474, *p* = 0.062) (Fig. 2).

Among those 25.293 cases, there were 19,464 cases undergoing delivery after the implement of NA. Among them, 3869 cases chose NA during labor, but 10 cases failed in epidural puncture, 2 cases showed detachment of epidural catheter during labor, and 238 cases required intrapartum CD based on medical indications. Finally, there were 3619 cases undergoing vaginal delivery with NA including 41 cases of accidental dural punctures by redone with proper placement, and 7563 cases without NA, so the overall rate of NA in vaginal delivery was 32.4% (3619/11182). Among those 3619 cases, the average VAS and NRS before the use of NA was 9.2, and then dropped to 3.1 after effective analgesia, which indicates the pain was significantly eased (*p*<0.001). Those who chose NA were satisfied with the effect of analgesia according to the results of questionnaire survey.

**Fig. 3:**
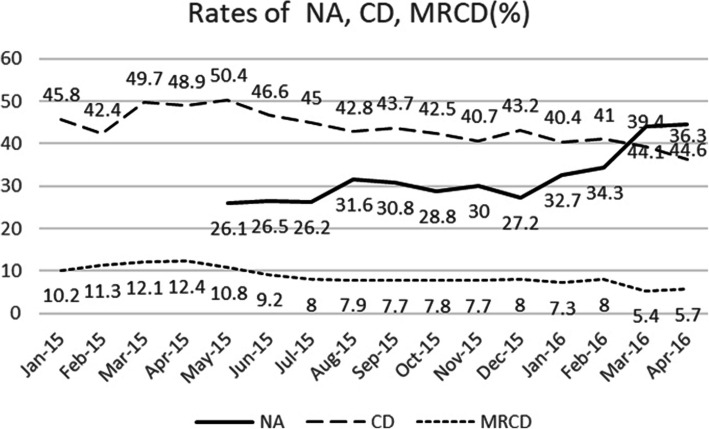
Rate of NA, CD, MRCD

The rates of CD, MRCD, and NA in 16-month observation are shown in Fig. 3 and Table 1. The results of Cochran- Armitage testing (Fig. 3) indicates that CD and MRCD rates were stable during the first 5 month in 2015(z = 3.080 and 1.114, *p* = 3.080 and 0.291>0.05), but the rates of CD and MRCD significantly decreased from 50.4% in May 2015 to 36.3% in April 2016 (z = 93.375, *p* = 0.000 *<* 0.001), and from 10.8% in May 2015 to 5.7% in April 2016 (z = 14.788, *p* = 0.000 *<* 0.001), respectively. Similarly, the rate of NA increased from 26.1% in May 2015 to 44.6% in April 2016(*p <* 0.001). Among the 16 months, there were a negative correlation between the rate of NA and rate of overall CD, r = − 0.782 (95%CI [− 0.948,-0.534], *p*<0.001), and between the utilization rate of NA and rate of MRCD, r = − 0.914 (95%CI [− 0.989,-0.766], *p*<0.001) .

**Table 1:** The relationships between the rate of NA and that of CD and MRCD during 16 months

		Rate of CD	(95%CI)	Rate of MRCD	(95%CI)
Rate of NA	r	−0.782	(−0.948 to − 0.534)	−0.914	(− 0.989 to − 0.766)
	*P*	<0.001		<0.001	

*NA* Neuraxial labor analgesia, *CD* Cesarean delivery, *MRCD* Maternal request cesarean delivery

The Pearson correlation was used

The change trend of episiotomy, operative vaginal delivery, PPH and neonatal asphyxia in women undergoing vaginal delivery.

The monthly rates of episiotomy, operative vaginal delivery, and PPH and neonatal asphyxia in women who undergoing vaginal delivery remained unchanged during the 16 months (4 months before and 12 months after the utilization of NA). Moreover, there was no correlation between NA and the rates of episiotomy, operative vaginal delivery, and PPH and neonatal asphyxia in women who underwent vaginal delivery (*p*>0.05) (Tables 2 and 3).

**Table 2:** The rates of NA, neonatal asphyxia, EP, ODV, PPH in vaginal delivery

Rate	Jan-15%	Feb-15%	Mar-15%	Apr-15%	May-15%	Jun-15%	Jul-15%	Aug-15%	Sep-15%	Oct-15%	Nov-15%	Dec-15%	Jan-16%	Feb-16%	Mar-16%	Apr-16%	X^2^	*p*-valua
NA	0	0	0	0	26.1	26.5	26.2	31.6	30.8	28.8	29.6	27.2	32.7	34.3	44.1	44.6	71.295	<0.001
neonatal asphyxia	1.4	1.3	1.2	1.3	1.3	1.3	1.3	1.2	1.0	1.4	1.0	1.1	1.3	1.1	1.1	1.3	0.705	0.401
EP	45.2	56.8	57.0	56.4	65.4	63.9	54.7	50.6	55.7	46.6	49.8	61.9	56.2	51.7	50.2	56.1	2.786	0.095
ODV	0.6	1.7	0.7	1.3	4.0	1.8	1.4	3.5	2.0	2.1	2.2	1.3	1.2	0.7	1.2	0.9	2.722	0.099
PPH	2.6	3.4	5.0	5.0	4.6	4.0	3.4	3.5	6.0	3.4	3.5	3.9	3.7	3.5	3.8	3.0	1.081	0.299

*NA* Neuraxial labor analgesia, *EP* Episiotomy, *ODV* Operation vaginal delivery (including forceps, vacuum and breech extraction), *PPH* Postpartum hemorrhage

Cochran- Armitage Trend Test was used

**Table 3:** The relationships between the rate of NA and that of neonatal asphyxia, EP, ODV, PPH in vaginal delivery women

		Rate of neonatal asphyxia	(95%CI)	Rate of EP	(95%CI)	Rate of ODV	(95%CI)	Rate of PPH	(95%CI)
Rate of NA	r	−0.369	(−0.670 to 0.000)	−0.017	(−0.483 to 0.391)	0.195	(−0.239 to 0.570)	−0.147	(−0.783 to 0.445)
	*p*	0.159		0.951		0.470		0.587	

*NA* Neuraxial labor analgesia, *EP* Episiotomy, *ODV* Operation vaginal delivery (including forceps, vacuum and breech extraction), *PPH* Postpartum hemorrhage

The Pearson correlation was used

The change trend of intrapartum CD, PPH and neonatal asphyxia in women who underwent CD.

From May 1st, 2015 to April 1st, 2016, there were 728 cases underwent intrapartum CD but only 8 cases underwent CD within the Labor & Delivery suite. From Tables 4 and 5, it can be found that the monthly rates of intrapartum CD, neonatal asphyxia and PPH in women undergoing CD remain unchanged during the 16 months of observation, and there was no correlation between the rate of NA and the rates of intrapartum CD, neonatal asphyxia, and PPH in women undergoing CD (*p* > 0.05).

**Table 4:** The rates of intrapartum CD, neonatal asphyxia, PPH in CD women

Rate	Jan-15%	Feb-15%	Mar-15%	Apr-15%	May-15%	Jun-15%	Jul-15%	Aug-15%	Sep-15%	Oct-15%	Nov-15%	Dec-15%	Jan-16%	Feb-16%	Mar-16%	Apr-16%	X^2^	*p*-valua
Intrapartum CD	9.2	8.4	8.4	8.2	7.8	8.0	8.7	8.6	9.1	9.5	8.3	10.2	8.6	9.6	9.1	8.4	0.415	0.519
Neonatal asphyxia	1.2	1.1	1.1	1.2	1.0	1.2	1.1	1.5	0.9	1.2	1.3	1.6	1.9	1.4	1.3	1.6	2.265	0.132
PPH	3.9	4.2	3.9	4.1	3.6	3.6	2.7	2.6	3.6	4.0	2.8	4.2	4.7	3.8	2.7	2.8	0.618	0.432

*CD* Cesarean delivery, *PPH* Postpartum hemorrhage

Cochran- Armitage Trend Test was used

**Table 5:** The relationships between the rate of NA and that of intrapartum CD, neonatal asphyxia, PPH in CD women

		Rate of intrapartum CD	(95%CI)	Rate of neonatal asphyxia	(95%CI)	Rate of PPH	(95%CI)
Rate of NA	r	0.198	(−0.194 to 0.555)	0.419	(0.121 to 0.684)	−0.494	(−0.795 to − 0.120)
	*p*	0.461		0.106		0.052	

*NA* Neuraxial labor analgesia, *CD* Cesarean delivery, *PPH* Postpartum hemorrhage

The Pearson correlation was used

## Discussion

At present, the high CD rate is a social problem in China. However, there are few hospitals conventionally carrying out NA during delivery and labor to meet the needs of parturient women [12]. Many Chinese people think labor pain is normal which is difficult to be avoided for parturient women especially nulliparous, so some of them choose CD, especially MRCD. Under the policy of one-child family taking effect from 1979, incorrect cognitions on delivery were prevalent in China, for example they thought the damage caused by episiotomy and that caused by CD were the same, CD might be less likely to affect the quality of sexual life than vaginal birth [13], and giving birth by selecting a particular date may be more safe [14]. The relative medical indications of CD vary all over China, such as pregnancy with severe shortsightedness which is believed to cause retinal detachment during labor in certain extreme condition, pregnancy by assisted reproduction techniques, primipara more than 35 years old [7, 15, 16]. Most obstetric centers in China cannot meet the increasing requirements of pregnant women, such as lacking the company of family members especially husband, analgesia, or emotional support in labor [3, 4]. In recent years, a few Chinese hospitals have started to control the unusually high CD rates with health education, painless delivery, doula delivery and psychological comforting and training programs for midwives and obstetricians [17]. In our birth center, with the help of NPLD program, NA has become available for women who request to relieve labor pain since May 1st, 2015.

There were several reports in the US back in the 1990s about the effects of a sudden increase in neuraxial analgesia rate on cesarean delivery rates, with almost all of them showing no effect [18, 19]. NPLD is a nongovernment project aiming to help Chinese parturient women and their health care providers about the safe and effective use of NA [7]. According to the data collected from Shijiazhuang Obstetrics and Gynecology Hospital where the project was first introduced in 2008, the rate of overall CD decreased from 40.5 to 33.6% in the period when the rate of neuraxial labor analgesia increased from 0 to 33.5% [20]. At the second Affiliated Hospital, Wenzhou Medical University, Wang Q found that as the labor epidural analgesia rate increased from 0 to 57%, the vaginal delivery rate increased, and cesarean delivery rate decreased by 3.5%, at the same time the rate of episiotomy and severe perineal injury were decreased [21]. Our results are in accordance with those observational studies on the use of NA with the help of NPLD [7, 20, 21]. In our study, before the implement of NA, the rates of overall CD and MRCD were stable and high from January 1st to May 30th 2015, but with the increase of usage of NA, both of them were dropped. At the early implementation stage of NPLD program, the rate of NA was the lowest (26.1%); while the rates of overall CD and MRCD were 50.36 and 10.76% in May 2015, respectively. As the rate of NA increased to 44.6% in April 2016, the rates of overall CD and MRCD decreased to 36.3 and 5.7% respectively. There was a negative correlation between the rate of NA with the rate of CD or MRCD, and there was a positive correlation between the rate of CD and that of MRCD. Our research was consistent with the other birth centers helped by NPLD in China, but inconsistent with that in the US back in 1990. The difference may be related to China’s family policy and the high rate of MRCD.

After implementing “one-child” policy for 30 years, the selective 2-child policy was announced in November 2013, which means that if one of the couples is the only child in her or his family, the couple will have the chance to have two children. China’s universal two- child policy was released in October of 2015 [22]. These policies may have an impact on the overall CD rate in China. Liao et al. [23] collected data from 6 hospitals in Hubei and Gansu province of China from 2013 to 2016 and found that the overall CD rate decreased from 45.1% during 1-child policy period to 40.4% during selective 2- child policy period, and further to 38.9% during universal 2- child policy period, which is consistent with the results of many other studies [24, 25]. Our study period was from May 1st 2015 to April 30th 2016, during which selective 2-child policy did not have a large influence on the overall CD rate. The monthly rate of parous from January 1st 2015 to April 30th 2016 in our birth center was still around 24.1% (6097/25293), which was not affected by the following universal two- child policy. From those analyses, we believed that the decrease of overall CD rate was mainly related to initiation of labor analgesia services.

Labor pain is probably the most severe pain that most women will endure in their lifetime. A neuraxial approach is accepted as the gold standard for intrapartum labor analgesia [26, 27]. The mixed drugs of ropivacaine and fentanyl administrated by PIEB pump is extremely safe and effective for labor delivery analgesia [26–28]. Our clinical trial demonstrated that the NA proposed by NPLD was effective, which reduced the delivery pain from average 9.2 to 3.1 according to VAS and NRS.

NA does not increase the risk of CD [7, 29], but its impact on operative vaginal delivery and other parturient safety outcomes is still controversial [30, 31]. Anwar S [30] conducted a quasi-experimental study and found that epidural analgesia did prolong the duration of second stage of labor and increased the instrumental delivery rate (58% vs 12%). Wassen MM [31] found the rate of epidural tripled increased from 7.7 to 21.9% over a 10-years span while the rates of CD and operative vaginal delivery did not change too much in the Netherlands. Our study showed that with the increase of NA rate, the rates of operative vaginal delivery remained nearly unchanged. As we all well know that it is difficult to study the effect of epidural analgesia on operative vaginal delivery rates under preconditions that are not double-blind [30, 31]. The reason why the instrumental delivery was increased may be probably due to that the presence of decent analgesia makes it much easier for obstetricians to apply forceps or vacuum [30]. In our birth center, the rate of instrumental delivery remained unchanged, which may be related to the low rate of instrumental delivery (1.6%, 234/14205) and still high rate of CD (43.8%, 11,088/25293) among the 16 months, most of instrumental delivery were instead by CD. At the same time, the rates of episiotomy, and PPH and neonatal asphyxia of vaginal delivery, and intrapartum CD, PPH, neonatal asphyxia of CD remained nearly unchanged before and after the use of NA in our birth center.

### Strengths and limitations of this study

The study shows the rate of NA, the rate of overall cesarean and the rate of MRCD in a large birth center of China with an annual capacity of 20,000newbornsbeing delivered.

The NPLD team helped to establish a neuraxial labor analgesia service in our hospital. The main limitation of this manuscript is that the findings are not so original that some other Chinese hospitals in conjunction with the NPLD program have reported similar results.

The data were selected from only one center, a tertiary- care teaching hospital in Hubei province, which cannot represent all hospitals from different geographical areas and in different levels in China.

The location of intrapartum CDs was moved to within the Labor & Delivery suite as part of the NPLD intervention, but there were only 8 cases underwent intrapartum CD within the Labor & Delivery suite to deal with maternal and fetal emergencies for extremely dangerous in our birth center, so it was difficult to analyze the relationship between the change in intrapartum CD and the location of CD(Labor & Delivery suite).

During the study period, China’s universal two-child policy was taking effect (2015.10), which may have some influence on the results.

## Conclusions

In conclusion, initiation of a 24-h labor analgesia services can relieve labor pain. Through a a16-month observation from May 1st 2015 to April 30th 2016, the rates of CD and MRCD were significantly reduced, which may be related to the increasing use of NA during delivery and labor by the help of NPLD.

## Supplementary information

**Additional file 1.** Team members of NPLD in our hospital.

## Data Availability

Access to the qualitative data should be given upon request to the corresponding author after taking any necessary precautions to safeguard participants’ privacy and confidentiality.

## Abbreviations

CD: Cesarean delivery
VD: Vaginal delivery
MRCD: Maternal request cesarean delivery
NPLD: No Pain Labor &Delivery
NA: Neuraxial labor analgesia
PPH: Postpartum hemorrhage
VAS: Visual analog scale
NRS: Numerical rating scale
