# Supplementary material for: The effect of initiating neuraxial analgesia service on the rate of cesarean delivery in Hubei, China: a 16-month retrospective study
Source: BMC Pregnancy Childbirth. 2020 Oct 12;20:613. doi: 10.1186/s12884-020-03294-z (PMC7552515; doi:10.1186/s12884-020-03294-z)
Supplement: Supplementary file 1 — Additional file 1. Team members of NPLD in our hospital. [file 12884_2020_3294_MOESM1_ESM.docx]

**Supplement file 1**

**Team members of NPLD in our hospital**

| **Name** | **Level of doctor** | **Department** | **Hospital** |
| --- | --- | --- | --- |
| Weike Tao* | Attending | Anesthesiology and Pain Management | University of Texas Southwestern Medical Center |
| Pamela Flood | Attending | Anesthesiology, Perioperative and Pain Medicine | Stanford University |
| Francis Stellaccio | Attending | Anesthesiology | Stony Brook University |
| Steven Shafer | Attending | Anesthesiology, Perioperative and Pain Medicine | Stanford University |
| Linden Lee | Resident | Anesthesiology and Pain Management | University of Texas Southwestern Medical Center |
| Brian Gelpi | Resident | Anesthesiology and Pain Management | University of Texas Southwestern Medical Center |
| Jeanne Sheffield | Attending | Maternal- Fetal Medicine; Gynecology and Obstetrics | Johns Hopkins University School of Medicine |
| Lisa Scheid | fellow | Neonatologist | University of Texas Southwestern Medical Center |
| Ho-yu Pan | nursing managers | Midwife | Duke university hospital |

* The team leader was born and studied in China, then immigrated to the United States. He was fluent in both Mandarin and English and familiar with Western standards of obstetric care.
